# Food As Medicine: Diet, Diabetes Management, and the Patient in Twentieth Century Britain

**DOI:** 10.1093/jhmas/jry008

**Published:** 2018-03-05

**Authors:** Martin D Moore

**Affiliations:** Wellcome Centre for Cultures and Environments of Health, University of Exeter, The Queen’s Drive, Exeter, EX4 4QH

**Keywords:** Diet, Diabetes, Patienthood

## Abstract

In classic accounts of the development of modern medicine in Europe and North America, the sick person is often portrayed as having a history of disappearance with the rise of the objectified body of the modern patient. To this account, sociologists and historians of medicine have added another for the period after 1950, in which the patient as subjective person “reappears” in medical discourse. However, despite histories of practice and identity revising narratives of disappearance, the patient’s reappearance has largely escaped further assessment. Using an analysis of dietary management in twentieth-century British diabetes care, this article challenges accounts of this reappearance in three ways. Firstly, it argues that discursive interest in the social and psychological aspects of care emerged earlier than suggested. Secondly, it grounds such interest in reconfigured institutional arrangements that were initially designed to rationalize care and improve efficiency. Finally, it argues that patients regularly exceeded the efforts of even an expanded management regime to normalize and regulate life. Food planning, preparation, and consumption continued to sit at the nexus of competing demands that mediated medical efforts to cultivate governable selves and bodies.

In recent decades, classic accounts of “the patient” in modern European and American medicine have come in for critique.^1^ The broad contours of these accounts still hold: that, with the rise of specific relations and practices of “hospital medicine” at the end of the eighteenth century, patient narratives became marginalized in medical discourse and training in favor of examinations, lesions, and discrete diseases, and that speaking patients were cast as unreliable witnesses to pathologies.^2^ Revisionist accounts, however, have asked what this meant for medicine in situ.^3^ They have argued that the passive patient of medical discourse was rarely seen during actual encounters, and have explored how patients had to learn novel relations to their modern bodies, their new “ignorance” the product of long negotiation and contact with changing institutions of medicine.^4^

Undertaking a study of the changing strategies for, and patient experiences of, dietary management in twentieth-century British diabetes care, this article asks what these insights might mean for analyses of “the reappearance” of the person in medical discourse.^5^ This work has suggested that medical discourse began to conceive of the sick person in new relational terms around the mid-twentieth century, as medicine placed disease in complex social settings.^6^ The subjectivity of doctors and patients became integral objects of investigation, and the speaking patient once more became an active member of the care team.^7^ To be sure, this new disposition did not mark a return to the world of Luigi Cornaro, outlined in Steven Shapin’s contribution to this issue. Though patients shared their experiences, and even used them to guide their care, their narratives were elicited solely as a means to more effective diagnosis and intervention. Moreover, this active patienthood was supposedly still a constructed subject position, the result of an expanded medical logic rather than a shared cultural heritage or broader social structures. In fact, older forms of practice also persisted within institutionalized medicine, and treating disease remained an important part of an expanded regulatory architecture.

As with other revisionist explorations of patienthood, this article does not deny that significant shifts in medical discourse and practice occurred. Rather, focusing on the dietetic management of diabetes in twentieth-century Britain, it re-examines the chronology of the changes outlined here, provides an alternative account of its possible cause, and explores the ways in which patients with diabetes negotiated the subject positions constructed for them. It argues that, although researchers and clinicians only drafted into use the tools of systematic study from the psychological and social sciences after the mid-century, medical texts discussing dietary plans across the early 1900s made frequent reference to the personal and social worlds of patients. It roots this shift, moreover, neither in an inherent necessity nor in new practices, as in the existing literature.^8^ Instead, it suggests that the development of diabetic out-patient clinics – grounded in shifting social and economic conditions – reoriented medicine’s gaze. Long-term and regular interactions with patients brought social and psychological problems into medicine’s spaces and management, and provided practitioners with initial opportunities to develop new concepts about the body, self, and society, which justified further change.

Finally, this article argues that patients regularly exceeded the efforts of medicine to manage and normalize their lives, moving beyond the subject positions crafted for them even after the extension of medical regulation in chronic diabetes care. Often a person’s sense of self was reoriented after diagnosis, and most patients and their networks made accommodation with medical direction to relieve severe physical symptoms, and restore economic normality.^9^ Nonetheless medicine’s expanded management strategies had limits.

Crucially, these limits can be seen most notably in dietary practice. Food planning, preparation, and consumption continued to sit at the nexus of competing demands – familial, economic, cultural, and social – that mediated medical efforts to cultivate governable selves and bodies.^10^ As such, though accepting the need for a complex routine of insulin injection and self-monitoring, only some patients felt the need to follow dietary direction to the letter. Others negotiated their dietary self-care around income, conflicting calculations of well-being, or working and family lives. Although a recognition of some of these issues formed the basis for an expanded medical discourse, many pressures on patients were still bracketed out and provided the basis of an interest in supposed “non-adherence.”^11^ Patient dietary practice, however, was not a form of resistance so much as a result of patients working creatively with medical knowledge and power.^12^ In looking at dietary management of diabetes in the twentieth century, then, this paper opens up an area in which medicine’s truths were complicated, and patients needed to compromise new biological needs with demands beyond medicine’s boundary of authority.

The work that follows is based primarily on a sampling of published medical sources and archived oral histories from a pre-existing collection – *Diabetes Stories* – produced by the Oxford Centre of Diabetes, Endocrinology and Metabolism between 2004 and 2007.^13^ Although extrapolating practice from publications carries risks, the mix of materials used enables an examination of shifts in medical discourse and glimpses at the work performed.^14^ Likewise, following Annmarie Mol, oral history testimonies have been productively read in a realist mode, whereby each individual is treated as their own ethnographer.^15^ Specific details are used, but concern over accuracy is in part obviated by a strong focus on general trends and recollections of routines of care.^16^

To outline the changes under discussion, this article will compare two periods of conceptual, practical, and institutional change. The first considers the development of “high-carbohydrate” diets in the late 1920s and early 1930s, relating changing perspectives on care to therapeutic innovation and experiences in new outpatient clinics. The second half of the essay turns to the explosion of interest in patient education and self-management during the late 1970s and 1980s. Technological innovations and institutional demands helped further develop perspectives that emerged in an earlier period. However, a comparison of changes in the 1920s and 1930s together with new systematic interest in issues of education and compliance nonetheless throws into sharp relief the expansion and continued limits of medical discourse and regulation across the whole period.

## Diabetes, High-Carbohydrate Diets, and Complications 1920s-1950s

During the first decades of the twentieth century, doctors with a special interest in diabetes largely reframed the condition in terms of metabolism.^17^ In Britain, as elsewhere, discussions of diabetes noted the cardinal symptoms of thirst, hunger, wasting, and excessive urination, but primarily came to focus upon the body’s incapacity to break down and utilize foodstuffs, and the resultant elevated blood glucose levels (hyperglycemia). Likewise, practitioners saw this accumulation of glucose as filtered out of the body through sugar in the urine (glycosuria), while implicating a disordered utilization of fat in the appearance of dangerous acid bodies.^18^ By the late 1910s, *The Lancet* could confidently assert that “diabetes is therefore a general disorder of the metabolism,” and though beliefs about the condition altered considerably over the following century, metabolic perspectives structured therapeutic strategies throughout the period under discussion.^19^

In light of such understandings, treatment in the early years of the twentieth century centered on controlling metabolism through the rational composition of diets. In the early 1900s, specialists recommended assessing a patient’s tolerance and severity, and subsequently designed a diet that replaced carbohydrates with protein and fats, as far as consistent with metabolic capacity.^20^ Into the 1910s, clinicians made adjustments to this position, recommending regulation of all inputs and increasingly emphasizing low calorie plans.^21^ Crucially, the successes of these efforts were largely monitored through the proxies of ketonuria and glycosuria.^22^ Such a strategy recognized the importance of metabolic control, while also respecting the impracticalities, cost, and possible professional implications of relying on blood analysis beyond in-patient care.^23^

The invention of insulin therapy altered the practice of management in complex ways.^24^ Scholars have begun to explore the exact mechanics of insulin’s introduction to Britain.^25^ Suffice it to say that insulin became widely available in 1924 and subject to the same working of the mixed economy of healthcare as other therapeutics: an individual’s dose would be tailored to their means or status.^26^

In therapeutic terms, insulin expanded the options available to clinicians, and during the 1920s some newly created specialist clinics registered around half of their patients as requiring some form of insulin treatment.^27^ Moreover, particularly for patients aged younger than fifty-five years at diagnosis, the drug played a crucial role in extending average, post-diagnosis, life expectancy.^28^ It took around a decade, however, before significant shifts were seen in dietary management strategies. Initially, doctors continued to prescribe weight-reducing diets to certain patients, and a number of elite doctors saw insulin as reinforcing strict dietary planning and adherence.^29^ And as a result of tailoring intake to a patient’s age, weight, and work demands, calorie intake for patients using insulin could range from 1400 calories to over 3,300 calories per day.^30^ Nonetheless, recommendations for carbohydrate load were generally kept low, and even the more generous doctors saw 70g of carbohydrate daily (equivalent to approximately three slices of bread today) as the fixed point of a “standard” diet.^31^

This situation began to change towards the end of the decade. Insulin injections enabled bodies to use carbohydrate more effectively, and clinical researchers and physiologists intensified suggestions that carbohydrate facilitated better management of ketones.^32^ Furthermore, the novel appearance of atherosclerosis and vascular disease in patients of long duration created concerns around cholesterol and fat intake.^33^ Beginning in North America and Europe, and then in the UK at the end of the 1920s, clinicians slowly increased the amount of carbohydrate allotted to diets for patients with diabetes.^34^ One debate between well-respected figures at the Royal Society of Medicine in 1931 indicated that on average this entailed doctors giving patients between 120g and 150g of carbohydrate, with 70g mentioned as a minimum of the range.^35^ Editorials in *The Lancet*, even mentioned allowances as high as 250g within a calorie-controlled diet.^36^

The rationale for these increases, however, went well beyond questions of metabolism. Clinical observation and medical teams’ exposure to patients’ experiences over the 1920s played a crucial role in such change. Insulin’s introduction to the UK was a catalyst for the formation of large out-patient clinics for diabetes follow-up, with such institutional forms providing new specialist functions for general hospitals.^37^ Interested doctors argued that successful insulin treatment required experience and organized access to laboratory testing, and specialist out-patient clinics provided a means of concentration. Generalist doctors and research scientists with recognized skills and facilities would receive referrals, and, proponents argued, could provide focused care, gain vital expertise, and treat growing numbers of patients more efficiently.^38^ They were, in many respects, the epitome of rationalized modern medicine, developed in a political culture of mass provision, and dependent upon a growing division of labor between members of hospital staffs.^39^

Crucially, however, now that clinicians were following patients over a long period, these doctors became exposed to the complaints and problems faced by their patients on a quotidian level. Whereas clinicians and research scientists removed in-patients from their social context for assessment and treatment before discharge, they now saw some out-patients consistently for months and years. Of course, as clinics grew, regularity of contact could break down.^40^ Nonetheless, this was not the case universally, and even discontinuous care brought broader problems into diabetes clinics. Thus, despite GPs providing care for some discharged patients, the rationalized and biomedically focused structures of modern medicine also provided hospital practitioners with exposure to the social worlds and experiences of patients.^41^ The spatial and institutional reorganization of the out-patient clinic, in this sense, provided a trigger for conceptual reorientation.

From the 1920s onwards, the leading figures in diabetes research, care and, publication were all situated in these clinics. Following this relocation, their discussions of treatment slowly altered, and they began to provide a socially-oriented rationale for dietary change. In particular, issues of anxiety, sociability and, palatability found their way into medical calculation. For instance, one editorial in *The Lancet* suggested that a key advantage of high-carbohydrate diets was that “the patient with diabetes need no longer make himself conspicuous by avoiding foods which contain carbohydrate.”^42^ Foods with particular cultural and political resonance, therefore, such as potatoes and bread, could be consumed, meaning more enjoyable social interactions and palatable meals.^43^ Equally, patients could avoid the feared social shaming that might come with divulging a diagnosis, with diabetes sometimes seen as infectious or as a disability.^44^ Furthermore, it is likely that dietary changes embodied other cultural values. For doctors and researchers, one of the initial reasons for trying to keep blood sugars within normal parameters was the belief that ersatz physiological regulation would secure better health.^45^ In some instances, the innovation of insulin reinforced medical belief that almost total control over metabolic balance could finally be achieved.^46^ For patients, by contrast, it is possible that eating a diet as close to “normal” as possible might also help minimize disruptions to their usual routine and sense of pre-diagnosed self.^47^ A diet approaching normality would thus offer social and psychological benefits even if not as beneficial in metabolic terms.

It was often in connection with higher-carbohydrate diets that doctors debated the criteria of therapeutic success, with biochemistry having to share space with other criteria in designing therapeutic programs. Doctors praised the diets for simplifying treatment for patients, allowing flexibility in practice, and facilitating patient productivity and social reintegration in line with concerns over national efficiency.^48^ Or, as one authority put it, the diets recognized that “men lived in homes and not in calorimetres.”^49^ It was also widely acknowledged that both insulin intake and diabetic diets could pose financial challenges to patients, and doctors considered carbohydrate diets cheaper than alternatives.^50^

Dietary discussions also played a significant role in prescriptive accounts of management produced for both doctors and patients. The pioneer of such guidance in Britain was R.D. Lawrence, a clinician who worked at King’s College Hospital, London, and a major figure in British diabetes care during the first half of the century. Lawrence was himself diagnosed with diabetes prior to the invention of insulin therapy, and suffered quite severely before early access to insulin saved his life.^51^ Following this transformative experience, Lawrence specialized in diabetes care and used his personal influence and connections with high-profile patients to fully equip a diabetic department at King’s.^52^ His clinic work (along with private practice and personal experience) encouraged him to think broadly about the difficulties faced by people with diabetes.

With regards to diet, Lawrence collaborated with biochemist colleagues to devise his own formula for dietary management. His line-ration scheme was widely used and adapted, combining tabularized chemical analyses of foodstuffs with clearly delineated portions of carbohydrate, fat and protein to produce daily dietary plans.^53^ Though Lawrence continued to advocate normal blood sugars and glycosuria-free urines as treatment aims, he accepted that they were neither always a possibility, nor the basis for a normal and happy life, for more severe patients.^54^ “If the patient desires it,” he wrote in 1931, “and has to use insulin on any diet, there is no logical reason for refusing more C. [carbohydrate] and insulin,” the effect of which would be to make the patient “happier mentally, if not much improved physically.”^55^ Such an approach also alleviated the need for patients to weigh and measure their proteins and fats, though the upper limit for these diets would still only be 120g carbohydrate.^56^ As with other practitioners, Lawrence made mental state and simplicity of life important therapeutic calculations.^57^

The line-ration scheme formed a central part of Lawrence’s broader attempts to promote his views and to assist patients adopt a role in maintaining their own health. He laid out this vision most clearly in his best-selling handbook for both practitioners and patients, *The Diabetic Life*. The work ran into seventeen editions over a forty-year period of publication, indicating an impressive readership and use.^58^ In this handbook, Lawrence proposed a number of rules for living, which – though sympathetic to challenges facing patients – sought to deeply reform a patient’s routine activities and social lives. Indeed, some advice was very direct and clear in this regard. Patients following guidance on weighing foodstuffs, or on the regularity and timing of insulin injection, for example, would clearly have to structure their eating routines around such advice. They might even have to reject invitations to friends’ houses where a “hostess…insists on offering “forbidden” foods on the plea that “once does not matter.””^59^

At its heart, then, rather than suggesting that patients were ostensibly “normal” and able to build management regimes around their own lives and preferences, the book sought to help patients live a normal life by educating them into accepting their condition. It sought to construct a new self for the patient through its promotion of a “diabetic creed,” one that would help them master their disease.^60^ In line with a growing political and cultural emphasis on personal hygienic regimen, Lawrence made clear that self-discipline and rational decision-making would be required.^61^ But, he promised, health would be the patient’s reward.

As a number of patient testimonies suggest, Lawrence’s book was widely used.^62^ Patients took advice from the work seriously, and along with guidance from their doctors, they recalled how it became the bedrock for deeply embedded patterns that came to structure their lives. If patients were children when diagnosed, parents often provided intermediary figures in care, operating as strict surveillance officers and enforcing lessons more strongly than would have been the case for adults.^63^ However, even once children became autonomous young adults, interviewees found themselves disciplined into certain actions. One interviewee (born 1925; diagnosed 1930), for instance, suggested that she came off the “Lawrence diet” at sixteen, having been diagnosed approximately aged five in 1930. Despite this, she recalls how since then “I always weighed my carbohydrate… I still do to this day. No particular reason, it’s just a long-standing habit.”^64^ In this case, we see how ingrained routines of practice could last well beyond initial training, becoming almost unconscious points around which life and regimen could be ordered.^65^

One might assume that this was the case for all patients. As David Arnold has suggested in the context of colonial and post-colonial India, insulin imposed a new form of government upon patients, centered on self-care practices set out by medical professionals.^66^ Yet, while it is clear that insulin injection and self-monitoring of urine were essential practices in this period, not all patients adopted the role afforded to them in relation to diet, eating, or even insulin injection. Whether on Lawrence’s plans or higher carbohydrate diets, patients worked with medical knowledge and pushed beyond the boundaries set in even more expansive medical discourse.

In part, this different stance related to differing family circumstances and competing social realities of early adult life. ^67^ And such determinants could go well beyond financial constraints.^68^ One interviewee, (born 1931; diagnosed 1939), suggested that though his father had read Lawrence’s book, other structural problems soon rendered attempts to follow the line ration method redundant.^69^ As he recalled, a strict regimen “was impossible…given how the school times varied, and given the fact too, I think, that my mother used to have great difficulty over coping with all the assortment of rations.” Pressures had been intensified during wartime, as their house became a “refuge for all sorts of people” during air raids, and his “poor mum did her nut over the cooking.” Similarly, once the interviewee had moved away from home to medical school, he stopped all weighing of food as it “wasn’t practical.” Thus, while he stuck rigidly to his insulin and self-monitoring regimen, he was too busy to watch his diet, which couldn’t “have been anything like as well controlled” as before. In such circumstances, appeals to self-control had little impact when non-medical structures influenced daily practices.^70^

Other patients also worked creatively with often-conflicting medical knowledge to suit their circumstances and outlooks, though less for economic and social reasons and more as a response to cultural differences. One interviewee (born 1926, diagnosed 1931), for example, discussed growing up in an expatriate community in Buenos Aires. Doctors there, he suggested, approached food differently than in England: “the Argentines were more liberal with food” and, unlike doctors in Stockport, “they seemed to suggest food intake…for different conditions.” Upon his return to England in the 1940s, the interviewee found medical advice “far too regimented” and conflicting with previous experience. As such, he continued to shape his diet and insulin intake in-line with how he felt, his previous experiences with food, and how he had been trained elsewhere.^71^

Thus, we see patients who did not always stick to the daily management brief laid as out by clinicians and handbooks. Medical discussion and regulation expanded to encompass social and personal aspects of patients’ lives, but patients could not, and did not always want to adhere to the restrictions imposed by care as much as by disease. Rather than resisting medical advice, they appropriated elements that worked (and could be fit around other demands), as well as working with conflicting recommendations to suit their previous experiences. Indeed, practitioners were clearly aware of such activity, and doctors complained as early as the 1900s that patients were liable to refuse or break diet once away from the “controlling influence of the medical man.”^72^ Into the post-war period, this interest in patient action and subjectivity found expression in research directed towards education and supposed poor compliance or adherence, reflecting a shift in medicine’s calculative self-reflexivity.^73^ Prior to this point, however, there was disagreement over whether to blame a lack of patient intelligence, or to adjust management strategies.^74^ These were early days in shifting medicine’s regime of truth beyond the inner-workings of the body, and systematic investigation had not commenced in such circumstances. This was to change in the 1970s and 1980s.

## Patient Education, Self-Management, and Improving Outcomes

Perhaps the biggest dispute to emerge within diabetological communities internationally after the 1940s related to the relationship between hyperglycemia and the onset of complications.^75^ At the extremes sat doctors who either believed that good metabolic control would be protective against complications (despite the cause not necessarily being known), and those who saw blindness, nerve damage, and cardiovascular and kidney disease as inevitabilities – merely the outcome of diabetes of long duration.^76^

In dietary terms, positions on this issue mapped onto discussions of what were known as “free diets.”^77^ Across the spectrum, most clinicians still prescribed calorie-controlled diets for overweight patients, believing that symptoms and metabolic disturbance would abate following weight reduction.^78^ For patients on insulin, however, proponents of free diets generally proposed a disregard for hyperglycemia and glycosuria in favor of a focus on ketones and avoiding hypoglycemia and loss of consciousness. Less restricted diets, argued advocates, were more mindful of broader criteria of success: a patient’s “weight, his ability to carry on, his well-being and his psychic outlook.”^79^

Free diets, however, gained little currency in Britain. Surveys suggest that clinicians generally adopted a middle-of-the-road position, balancing the aims of metabolic control with the limitations posed by urine tests and insulin injections.^80^ Patients, too, rarely mentioned changing their diets based on shifting advice from their medical advisers. By the later 1970s and 1980s, doctors were becoming more convinced that normal glycemia offered protection against complications. New epidemiological studies indicated that, although not inevitable, the risk of retinal damage was significantly enhanced in populations with higher average glycemia results.^81^ Clinicians often found this evidence persuasive – if not conclusive – and new monitoring technologies appeared to make metabolic control more achievable than ever.^82^ Mobile blood testing units offered greater certainty to patient self-testing, and tests for glycosylated hemoglobin (HbA1c) provided clinicians an average reading of glycemia levels over a 120-day period.^83^

Despite this growing certainty about the relationship between control and complications, late-twentieth century dietary advice was not straightforward. Over the post-war period, medical and political interest in the chronic problems that might follow “over-nutrition” had grown considerably, and epidemiologists, clinicians and laboratory scientists worked across various sites to devise theories about the benefits and dangers represented by specific nutritional elements.^84^ However, despite decades of research, there was little agreement about the possible effects of dietary intervention. This lack of consensus was expressed from within the new policy arenas and health education work of post-war public health.^85^ In terms of diabetes, advice from the British Diabetic Association was forthcoming in the mid-1980s.^86^ Yet, broader disputes still translated into continued debate about the role of dietary fats, fiber and other metabolic markers in the onset and prevention of certain complications.^87^

As they had earlier in the century, patients continued to work creatively with medical knowledge, adjusting diet in light of traditional familial dynamics.^88^ One interviewee, for instance (born 1931, diagnosed in Birmingham in 1964), outlined how his wife initially cooked two sets of meals after his diagnosis – one for him and the other for the family. Feeling guilty about the extra labor this implied for his wife, though without deconstructing the breadwinner model, the interviewee drew upon his training and told his wife to “make the [family] meal as usual and I will eat what I think my rations would come to.” Although domestic arrangements freed the interviewee from certain duties, his new health status generated conflicting goals and feelings – the first was adherence to dietary rules believed to benefit his body, the second was a desire for more harmonious domestic living. In the end, the interviewee found a compromise. Though for decades unaffected by subsequent changes in dietary advice, this settlement required working productively with his training rather than resisting it. Compromising nonetheless meant that he exceeded the role *traditionally* afforded to patients, even in diabetes’ expanded medical discourse.^89^

Strikingly, such decision-making became a focus of interest in the later decades of the twentieth century, with practitioner and patient in medical discourse looking rather different to their counterparts of the 1930s. In-line with assessments of medical discourse outlined in the introduction, the communication and subjectivity of doctors became the object of sustained study and criticism in assessments of dietary behavior after the 1960s.^90^ Likewise, while during the 1930s the mental effects of treatment began to feature as important points of dialogue and calculation, later discussions within specialist publications regularly focused on the psychological and social strains of diabetes and its management. Pioneers in the field even suggested that “the identification and prevention of social and emotional problems are arguably of more importance in preventing complications than new methods of insulin delivery,” and such figures were at the forefront of experiments with peer and expert support.^91^

The new patient – one with psychological, social and emotional issues that required careful management – could also be seen in terms of educational programs. During the 1920s and 1930s, even the most sympathetic doctors wrote of education as a straightforward transmission of knowledge. Once patients had been informed, they could combine their knowledge with professional advice to achieve a degree of self-care. By the close of the twentieth century, however, some doctors were discussing patient training in light of educational theories. Education was now subject to systematic assessment, and geared towards providing not just knowledge but “*the possibility* for each person’s development into an active (thinking) diabetic.”^92^ Education would provide motivation, but be weighed against “type of diabetes, age, personality, intelligence, education, common sense, interest in learning, family support, and associated diseases.”^93^ And as such, doctors should aim “to provide the possibility for the best possible control *in the circumstances of this particular patient*, so as to avoid hospital admissions and to minimise complications by a programme which *neither distorts his life nor impedes reasonable expectations*.”^94^

Indeed, according to those who followed this conceptualization, as a thinking person who should adjust therapy in-line with other demands on their life, patients’ dietary choices should be respected. This was particularly the case where disputes concerned potential benefits. As one article suggested in relation to fiber “for many patients, the quality of life associated with having eggs and bacon for breakfast rather than beans or a high fibre cereal is worth a possible modest reduction in life expectancy,” and such decisions were perfectly legitimate.^95^ A minority of interlocutors on this point were clearly influenced by the changing nature of discourses on “patient consumerism” in this period, which provided a new lens through which “active patients” could be constructed.^96^ One writer, for instance, compared doctors to a waiter who should merely provide options for patients to choose from.^97^ Others, however, combined socio-psychological medical discourse with traditional clinical concerns. They proposed that a doctor laying out options without recommendations was “guilty of shirking his duty if not of malpractice.”^98^ A patient was free to disregard recommendations, but such recommendations should still be made.

As the literature suggests, this new medical vision sought to push medicine’s truth claims into new areas – even if only to establish thresholds of legitimacy – and sought to regulate psychological and social realms more effectively. Yet, there were clearly limits to this expanded medical vision. Firstly, only the doctors most influenced by sociological techniques ever discussed broader structural issues that influenced how patients prepared and consumed food.^99^ Most recommendations for improving dietary management continued to focus on education, motivation, communication, and surveillance.^100^ This was despite patients being anxious about employment and workplace demands, and using knowledge of the self to adjust to these and broader cultural demands about the body.

For example, though she suggested having faced no pressure other than her own “vanity,” one interviewee (born 1940, diagnosed 1967) recalled how she wished to remain “thin” following her diagnosis – an embodied ideal frequently demanded of young women during the latter half of the twentieth century.^101^ Her diet was arranged accordingly, and she managed to keep within acceptable levels of metabolic control for much of her life. However, the interviewee nonetheless recalled periods of her life when stresses at work and home made balancing her diabetes a challenge. The time and energy demands of her role in the catering trade, for instance, regularly caused the interviewee to experience hypoglycemia, as breaks for refreshment were rare. Similarly, she recalled how, in an office job later in life, “I was very frightened if I was in any meeting or anything that I might go hypo, it made me nervous so I would always make sure I [her blood glucose] was higher rather than lower.” Indeed, though sticking to her diet was crucial for this interviewee, she nonetheless recalled how her life became structured around employment: “I had to keep a roof over my head, pay the mortgage. I didn’t want to lose my home, or my children’s home… And that was what mattered… I had to be alright for work.”^102^ Such decisions potentially resulted in worsened health, but were the only choices that the interviewee had. They were also choices that practitioners across the century rarely acknowledged in writing, and were taken in an environment that rarely became subject for campaigns to change.

Secondly, discussions about metabolic control over the last decades of the twentieth century continued to explain poor blood glucose control of in terms patients’ lack of information, motivation, poor teaching, and simple non-adherence, raising questions for service delivery.^103^ On the one hand, of course, the very existence of research into poor control and improving education and support was a symbol of medicine’s practical limits; such activity implicitly acknowledged that existent regimes were unable to make all patients adhere to agreed programs, and that patients continued to operate in ways not sanctioned by medicine’s regulatory machinery.

On the other, this research also tended to generate responses that reaffirmed medicine’s conceptual boundaries. Broader constraints potentially limiting patient activity were largely set aside for a focus on individual patients and practitioners. Medical teams thus redoubled suggestions to assess patient knowledge and attitudes through surveys, to experiment with new techniques and technologies of education, and to reorder sites and organization of health care delivery to allow greater focus on patient training, support and oversight.^104^ In terms of the latter, the diabetic clinic became the object of criticism, with hospital doctors and general practitioners alike suggesting that such institutions were too overcrowded, the result of inadequate resources relative to patient load. In their stead, reformers suggested that patients would benefit from coordinated general practice shared-care schemes, as well as from diabetes centers that specialized in ongoing advice and comprehensive surveillance.^105^ Recognizing other drivers for reform, such institutions were also described as money-savers, preventing expensive hospital care resulting from complications.^106^

By contrast with the first half of the century, then, experts of this later period were becoming much more systematic in their approach to producing responsible patients, retraining medicine’s increasingly self-reflexive techniques of assessment onto the patient to promote self-care.^107^ And such efforts often had considerable success, affecting positive changes. A large number of patients felt better able to make decisions and gain control of their care, as well as improving their metabolic outcomes. Furthermore, the latter seemed to translate to other improvements (such as fewer amputations, or increased weight loss, at least in the short term) and money was saved in those areas with the most effective reallocation of resources.^108^ Yet, at the same time, such efforts were also a reflection of both medicine’s expansion into new realms of truth since the early 1900s, and its continued conceptual and practical boundaries in relation to patients’ lives.

## Conclusion

Reflecting on her husband’s life and his experiences with diabetes, one interviewee recalled how, in conversations, he had spoken “about him going to dinners,” and how “Dr Lawrence [his employer] would just go into the gents and inject himself straight through his trousers… He drank, and did everything that you were not supposed to… And it’s certainly the model my husband has followed.”^109^ Despite the extending reach of medical discourse and regulation over the twentieth century, this interviewee’s husband rarely followed the dietary advice laid down for him. He did not necessarily resist this advice, nor find it malicious in intent. For example, he undertook self-monitoring tests and injected therapeutics that very much kept him well. Instead, he worked productively with medical knowledge, and adjusted his insulin and intake to suit his activities with family and friends, and the demands of his work.

This particular patient was like many others diagnosed with diabetes in Britain during the twentieth century, who were subjects in self-management regimes and dietary plans. The remit of medicine’s claims to knowledge undoubtedly expanded over the years following 1900, with considerable influence on the lives of its objects and subjects. Following spatial and technical innovations at the turn-of-the-century, the inclusion of what might be termed social and psychological aspects of care into medical discourse marked considerable shifts from previous focus on the internal workings of the isolated, standardized body. Likewise, after the 1970s, doctors and researchers formalized programs for educating and normalizing errant patients, signifying a connected shift in the regulatory apparatus of diabetes care.

And yet, though patients with diabetes in the twentieth century consistently found their lives and selfhoods reoriented, they also consistently exceeded the limits of new subject positions that had been crafted for them. This is not to say that patients were resistant to a malignant medicine, or that their agency resulted from some innate personhood. Where diet was concerned, for instance, patients could not escape the cultural and economic structures into which food production, preparation, and consumption had been embedded. Rather, such activity merely confirmed that medicine’s efforts to govern autonomous patients had definite limitations.

When writing histories of patients and medical discourse in twentieth-century Britain, America, or Europe, then, we must be wary of the figure of the reappearing subjective person. Discursive constructions and medical encounters, though clearly functioning in interconnected ways, have proven themselves frequently discordant. The place of the sick person in twentieth-century medical practice is testament to this discordance. By paying attention to both discourse and practice – patients and patienthood – as well as to the ways in which medical professionals themselves have calculated and reacted to social realities, we are able to gain new traction on medicine’s power and functions in this period.

As Steven Shapin has shown in his contribution, diet provides an ideal entry point through which to explore questions of individuality, expertise and medical authority. Diet has long held a central place within debates and practices of health, and retained its centrality to schemes for bodily and disease self-management into the twentieth-century.^110^ However, whereas early modern disputes between lay persons and doctors were structured by a shared dietetic culture and medical marketplaces, by the twentieth century, dietetics – in diabetes, and in general – had become the focus for bodies of scientific knowledge produced by experts.^111^ Although patients often had a sense of what worked for them, their reworking of medical direction did not tend to emerge out of this expertise (as for Carnaro). Rather decisions about what patients should eat were influenced by the cultural embeddedness of food and the social pressures surrounding its consumption.

## Acknowledgements

Once again, I am deeply indebted to Mark Jackson, Gareth Millward, and Harriet Palfreyman for the comments passed on this paper, as well as to Roberta Bivins and the Balance Group at the University of Exeter for their guidance and advice. This work was generously supported by the Wellcome Trust [Grant Number: 100601/Z/12/Z].

## Footnotes

1 Erwin Ackernecht, *Medicine at the Paris Hospital, 1794-1848*, (Baltimore: Johns Hopkins Press, 1967); Michel Foucault, [translated by A.M. Sheridan Smith], *The Birth of the Clinic: An Archaeology of Medical Perception*, (London: Routledge, [1973] 2010); Nicholas Jewson, “The Disappearance of the Sick Man from Medical Cosmology, 1770-1870,” *Sociology* 10 (1976): 225-44.

2 David Armstrong, “The Patient’s View,” *Social Science and Medicine* 18 (1984): 737-44; Mary E. Fissell, “The Disappearance of the Patient’s Narrative and the Invention of Hospital Medicine,” in *British Medicine in an Age of Reform*, ed. Roger French and Andrew Wear, (London: Routledge, 1991), 92-109; Jonathan Gillis, “The History of Patient History since 1850,” *Bulletin of the History of Medicine*, 80 (2006): 490-512; Graham Mooney, “Diagnostic Spaces: Workhouse, Hospital and Home in Mid-Victorian London,” *Social Science History* 33 (2009): 357-90.

3 Judith Walzer Leavitt, “Medicine in Context: A Review Essay of the History of Medicine,” *American Historical Review* 95 (1990): 1471-84.

4 Nancy M. Theriot, “Negotiating Illness: Doctors, Patients and Families in the Nineteenth Century,” *Journal of the History of Behavioural Sciences* 37 (2001): 349-68; Sally Wilde, “The Elephants in the Doctor-Patient Relationship: Patients’ Clinical Interactions and the Changing Surgical Landscape of the 1890s,” *Health and History* 9 (2007): 2-27.

5 Flurin Condrau, “The Patient’s View Meets the Clinical Gaze,” *Social History of Medicine* 20 (2007): 525-40.

6 David Armstrong, *The Political Anatomy of the Body: Medical Knowledge in Britain in the Twentieth Century*, (Cambridge: Cambridge University Press, 1983).

7 William Ray Arney and Bernard J. Bergen, *Medicine and the Management of Living: Taming the Last Great Beast*, (Chicago: University of Chicago Press, 1985); Thomas Osborne, “Power and Persons: On Ethical Stylization and Person-Centred Medicine,” *Sociology of Health and Illness*, 16 (1994): 515-35.

8 For instance: Arney and Bergen, *Medicine and the Management of the Living*, 62-8.

9 Dick Willems, “Managing One’s Body Using Self-Management Techniques: Practicing Autonomy,” *Theoretical Medicine and Bioethics* 21 (2000): 23-38.

10 Deborah Lupton, *Food, the Body, and the Self*, (London: SAGE, 1996 [1998]); Steven Shapin, “How to Eat Like a Gentleman: Dietetics and Ethics in Early Modern England,” in *Right Living: An Anglo-American Tradition of Self-Help Medicine and Hygiene*, ed., C. Rosenberg, (Baltimore: Johns Hopkins University Press, 2003): 21-58; Frances Steel, “A Source of Our Wealth, Yet Adverse to Our Health? Butter and the Diet-Heart Link in New Zealand to c.1990,” *Social History of Medicine* 18 (2005): 475-93; Tripp Rebrovick, “The Politics of Diet: ‘Eco-dietetics,’ Neoliberalism, and the History of Dietetic Discourses,” *Political Research Quarterly* 68 (2015): 678-89.

11 Jeremy A. Greene, “Therapeutic Infidelities: ‘Noncompliance’ Enters the Medical Literature, 1955-1975,” *Social History of Medicine* 17 (2004): 327-43.

12 Willems, ”Managing One’s Body Using Self-Management Techniques,” 23-38; Helen K. Valier and Roberta Bivins, “Organization, Ethnicity and the British National Health Service,” in *Innovations in Health and Medicine: Diffusion and Resistance in the Twentieth Century*, ed. Jennifer Stanton, (London: Routledge, 2002), 37-64.

13 The collection contains interviews with patients, family members and professionals, covering the 1920s to the early 2000s. All contributors have agreed for their testimony to be used in research and publication. For terms of use, see Anon, “Terms of Use for Diabetes-Stories,” *Diabetes Stories*. Available at: http://www.diabetes-stories.com/terms.asp. Accessed on Feb 29 2016. All material has been checked to avoid damaging reputations or misrepresenting the views expressed by interviewees: Valerie Raleigh Yow, *Recording Oral History: A Guide for the Humanities and Social Sciences*, 3^rd^ Edition, (London: Rowman & Littlefield, 2015), 159-84.

14 John Harley Warner, *The Therapeutic Perspective: Medical Practice, Knowledge and Identity in America, 1820-1885*, (Cambridge, Mass.: Harvard University Press, 1986).

15 Annemarie Mol, *The Body Multiple: Ontology in Medical Practice*, (Durham: Duke University Press, 2002).

16 Alistair Thomson, “Four Paradigm Transformations in Oral History,” *Oral History Review* 34 (2007): 49-70. The material would also provide excellent material for a study in the social construction of memory. Nonetheless, when read together critically with other sources the material used can still offer a useful view onto personal experience.

17 L. Sawyer and E.A.M Gale, “Diet, Delusion and Diabetes,” *Diabetologia* 52 (2009): 1-7.

18 “Treatment of Diabetes,” in *A Manual of Medical Treatment and Clinical Therapeutics*, vol. 2, ed. I. Burney Yeo, Raymond Crawfurd and E. Farquhar Buzzard, 5^th^ Edition (London: Cassell and Co., 1913): 519-48.

19 “Modern Views on Diabetes,” *The Lancet* 192 (1918): 49.

20 This was achieved through trial and error, adjusting diet until glycosuria increased and then disappeared: “Diabetes Mellitus,” in *An Index of Treatment by Various Writers*, ed. Robert Hutchison and H. Stansfield Collier, 1^st^ Edition, (Bristol: John Wrght and Co., 1907): 207-10.

21 “Alimentary Rest in Diabetes” *The Lancet* 188 (1916): 25-6.

22 P.J. Cammidge, “The New Dietetic Treatment of Diabetes,” *The Lancet*, 190, (1917): 524.

23 Christopher Lawrence, *Rockefeller Money, The Laboratory and Medicine in Edinburgh, 1919-1930: New Science in an Old Country*, (New York: University of Rochester Press, 2005): esp. 269-302.

24 Michael Bliss, *The Discovery of Insulin*, 25^th^ Anniversary Edition, (Chicago: University of Chicago, 2007); Chris Feudtner, *Bittersweet: Diabetes, Insulin and the Transformation of Illness*, (Carolina: Carolina University Press, 2003).

25 Robert Tattersall, *Diabetes: The Biography*, (Oxford: Oxford University Press, 2009): 52-79; Jonathan Liebenau, “The MRC and the Pharmaceutical Industry: The Model of Insulin,” in *Historical Perspectives on the Roles of the MRC: Essays in the History of the Medical Research Council of the United Kingdom and its Predecessor, the Medical Research Committee, 1913-1953*, ed., Joan Austoker and Linda Bryder, (Oxford: Oxford University Press, 1989): 163-80.

26 This may have included discontinuing insulin altogether: C.J.C Earl, “The Treatment of Diabetics as Hospital Out-Patients,” *British Medical Journal* 1 (1927): 833.

27 Ibid. Other clinics initially dealt with mainly dietary patients, while later some recorded an average of 75 percent insulin-taking patients. Cf: “Scotland,” *The Lancet*, 140 (1924): 461; G.F. Walker, “Reflections on Diabetes Mellitus: Answers to a Questionary,” *The Lancet*, 262 (1953): 1329-32.

28 George Graham, “Diabetes Mellitus: A Survey of Changes in Treatment During the Last Fifteen Years,” *The Lancet* 232 (1938): 1-7, 2. American assessments suggested average post-diagnosis life expectancy increased from 4.9 years to 10.4 years between 1897-1914 and 1930-6, with greatest increases for the young: Elliott P. Joslin, “A Half-Century’s Experience in Diabetes Mellitus,” *British Medical Journal* 1 (1950): 1095.

29 George Graham, “On the Present Position of Insulin Therapy,” *The Lancet* 204 (1924): 1265-6.

30 Ibid; R. Pybus and D. Murray Lyon, “A Table of Standard Diets for Use in Insulin,” *British Medical Journal* 2 (1924): 326-9.

31 A. Clarke Begg, “A Standard Diet for Insulin Treatment,” *The Lancet* 203 (1924): 516-7.

32 H.P. Himsworth, “Recent Advances in the Treatment of Diabetes,” *The Lancet* 218 (1931): 978-9.

33 S.C. Dyke, “Increased Carbohydrate in the Treatment of Diabetes Mellitus,” *The Lancet* 219 (1932): 980.

34 “Diet in Diabetes,” *The Lancet* 220 (1932): 1116.

35 “Royal Society of Medicine,” *The Lancet* 217, (1931): 351-3.

36 “Diet in Diabetes,” *The Lancet* 217 (1931): 142.

37 George Weisz, *Divide and Conquer: A Comparative History of Medical Specialization*, (Oxford: Oxford University Press, 2006). Cf diabetes specialization in the US: Feudtner, *Bittersweet*.

38 “Scotland,” 461.

39 “The Management of Diabetic Out-Patients,” *The Lancet* 231 (1938): 509; Steve Sturdy and Roger Cooter, “Science, Scientific Management and the Transformation of Medicine in Britain, c.1870-1950,” *History of Science* 34 (1998): 421-66.

40 Cf: Earl, “The Treatment of Diabetics as Hospital Out-Patients,” 831-3; Hugo Droller, “An Outbreak of Hepatitis in a Diabetic Clinic,” *British Medical Journal* 1 (1945): 624.

41 A. Clarke Begg, *Insulin in General Practice: A Concise Clinical Guide for Practitioners*, (London: William Heinemann (Medical Books), 1924).

42 “Diet in Diabetes,” 142.

43 “Disabilities: 21. Diabetes,” *The Lancet* 253 (1949): 116.

44 Ibid; Oral History Interview with Mo Linton performed by the University of Oxford, *Diabetes Stories*, 05/09/2007: http://diabetes-stories.com/transcript.asp?UID=73. Accessed 16 January 2016.

45 “Diet in Diabetes,” 1116. Certain doctors even suggested that achieving consistently normal blood sugars was correct because it was physiological: Graham, “The Present Position of Insulin Therapy,” 1265.

46 Chris Feudtner, “The Want of Control: Ideas, Innovation, and Ideals in the Modern Management of Diabetes Mellitus,” *Bulletin of the History of Medicine* 69 (1995): 66-90.

47 On chronic illness and the self: Kathy Charmaz, *Good Days, Bad Days: The Self in Chronic Illness and Time* (New Brunswick: Rutgers University Press, 1991).

48 “Royal Society of Medicine,” 351-3; Sturdy and Cooter, “Science, Scientific Management and the Transformation of Medicine in Britain,” 421-66.

49 “Royal Society of Medicine,” 352.

50 Himsworth, “Recent Advances in the Treatment of Diabetes,” 978.

51 James G.L. Jackson, “R.D. Lawrence and the Formation of the Diabetic Association,” *Diabetic Medicine* 13 (1996): 9-22.

52 Ibid.

53 Wilfrid Oakley, “Diabetes Mellitus,” in *The British Encyclopaedia of Medical Practice*, vol.3, ed. Sir Humphrey Rolleston, 1^st^ Edition, (London: Butterworth & Co, 1936): 653-6.

54 R.D. Lawrence, *The Diabetic Life: Its Control by Diet and Insulin, A Concise Practical Manual For Practitioners and Patients*, 6th Edition, (London: J.A. Churchill, 1931): 67-71.

55 Ibid, 63.

56 “Royal Society of Medicine,” 351-2

57 Ibid, 351-3.

58 R.D. Lawrence, *The Diabetic Life: Its Control by Diet and Insulin and Oral Treatment by Sulphonyl-Ureas, A Concise Practical Manual*, 17th Edition, (London: J.A. Churchill, 1965).

59 Lawrence, *The Diabetic Life,* 1^st^ Edition, 115.

60 Ibid, iii-iv.

61 Ina Zweiniger-Bargielowska, *Managing the Body: Beauty, Health and Fitness in Britain, 1880-1939*, (Oxford: Oxford University Press, 2010).

62 Oral History Interview with Richard Fawkes performed by the University of Oxford, *Diabetes Stories*, 23/11/2004: http://www.diabetes-stories.com/transcript.asp?UID=28. Accessed 15 January 2016; Oral History Interview with Margaret Elliott performed by the University of Oxford, *Diabetes Stories*, 07/12/2004: http://www.diabetes-stories.com/transcript.asp?UID=33. Accessed 15 January 2016.

63 Ibid; Oral History Interview with Dr Rev. Francis Andrews performed by the University of Oxford, 27/05/2004: http://www.diabetes-stories.com/transcript.asp?UID=8. Accessed 15 January 16. Also: Feudtner, *Bittersweet*; James W. Wheless, “History of the Ketogenic Diet,” *Epilepsia*, 49 (2008): 3-5.

64 Interview with Margaret Elliott performed by the University of Oxford.

65 Willems, “Managing One’s Body Using Self-Management Techniques.”

66 David Arnold, “Diabetes in the Tropics: Race, Place and Class in India, 1880-1965,” *Social History of Medicine* 22 (2009): 258.

67 As in earlier centuries, food choices were influenced by multiple factors: Paul Lloyd, “Nutritious Foods and Consumption Choices in the Early Modern Period,” *Social History of Medicine* 24 (2011): 161-5.

68 Oral History Interview Margaret Elliott.

69 Oral History Interview with Dr Rev. Francis Andrews.

70 These problems continue today: Annmarie Mol, *The Logic of Care: Health and the Problem of Patient Choice*, (London: Routledge, 2008).

71 Interview with Richard Fawkes.

72 W. Hale White, “An Address on Glycosuria,” *The Lancet* 183 (1914): 372.

73 Doctors used both terms to discuss patients following medical advice**:** Greene, “Therapeutic Infidelities,” 327-43.

74 On intelligence: D.M. Lyon, “Prognosis in Diabetes Mellitus,” *The Lancet* 199 (1922): 1045.

75 Christiane Sinding, “Flexible Norms? From Patients’ Values to Physicians’ Standards,” in *Histories of the Normal and the Abnormal: Social and Cultural Histories of Norms and Normativity*, ed. Waltraud Ernst, (London: Routledge, 2006), 229-32.

76 Derrick Dunlop, “Are Diabetic Degenerative Complications Preventable?,” *British Medical Journal* 2 (1954): 383-5.

77 Sinding, “Flexible Norms?,” 233-5; Tattersall, *Diabetes*, 85-90.

78 George R.W.N. Luntz, “The Diet in Diabetes,” *British Medical Journal* 2 (1943): 21.

79 R.H. Micks, “A Two-Stage Method of Treatment in Diabetes,” *British Medical Journal* 2 (1944): 784-6.

80 Walker, “Reflections on Diabetes Mellitus,” 1329-32.

81 H. Al Sayegh, and R.J. Jarrett, “Oral Glucose-Tolerance Tests and The Diagnosis of Diabetes: Results of A Prospective Study Based on the Whitehall Survey,” *The Lancet* 314 (1979): 431-3.

82 K.M. West, “Hyperglycaemia as a Cause of Long-Term Complications,” in *Diabetic Complications*, ed. Harry Keen and John Jarrett, 2^nd^ edition, (London: Edward Arnold, 1982): 13-18.

83 Boas Gonen, Hyman Rochman, Arthur H. Rubenstein, Sonia Tanega and David L. Horwitz, “Haemoglobin A1: An Indicator of The Metabolic Control of Diabetic Patients,” *The Lancet* 310 (1977): 734-7. R. Worth, P.D. Home, D.G. Johnson, J. Anderson, Linda Ashworth, Jaqueline M. Burrin, D. Appleton, C. Binder, K.G.M.M. Alberti, “Intensive Attention Improves Glycaemic Control in Insulin-Dependent Diabetes without Further Advantage from Home Glucose Monitoring: Results of a Controlled Trial,” *British Medical Journal* 285 (1982): 1233-40.

84 John Cleave, *The Saccharine Disease: Conditions Caused by the Taking of Refined Carbohydrates, such as Sugar and White Flour*, (Bristol: John Wright & Sons, 1974); H.C. Trowell and D. Burkitt, eds, *Western Diseases: Their Emergence and Prevention*, (London: Edward Arnold, 1981).

85 Mark Bufton and Virginia Berridge, “Post-War Nutrition Science and Policy Making in Britain c. 1945-1994: The Case of Diet and Heart Disease,” in *Food, Science, Policy and Regulation in the Twentieth Century: International and Comparative Perspectives*, ed., D. Smith and J. Phillips, (London: Routledge, 2000): 207-20.

86 M.E.J. Lean and W.P.T. James, “Prescription of Diabetic Diets in the 1980s,” *British Medical Journal* 292 (1986): 723-5.

87 J.I. Mann, “What Carbohydrate Foods Should Diabetics Eat?,” *British Medical Journal* 288 (1984): 1025-6.

88 Alice Julier, “Family and Domesticity,” in Amy Bentley, ed., *A Cultural History of Food in the Modern Age* (London: Bloomsbury, 2013): 145-63.

89 Oral History Interview with George Saunders performed by the University of Oxford, 14/05/2004: http://diabetes-stories.com/interview.asp?UID=6. Accessed 23 January 2016.

90 “Diabetes Mellitus – Obesity and Dietary Management,” *British Medical Journal* 2 (1972): 706-8.

91 R.B. Tattersall and J.G.L. Jackson, “Social an Emotional Complications of Diabetes,” in *Complications of Diabetes*, 271; M.O. Aveline, D.K. McCulloch and R.B. Tattersall, “The Practice of Group Psychotherapy with Adult Insulin-Dependent Diabetics,” *Diabetic Medicine* 2 (1985): 275-82.

92 Quoted in: M.I. Drury, “Diabetic Education: A Physician’s View – Who, What, Where, When and How,” *Diabetic Medicine* 1 (1984): 233. Original emphasis.

93 Ibid. 234.

94 Ibid. 233. Original emphasis.

95 Cited in: Robert Tattersall and Peter Mansell, “Fibre in the Management of Diabetes: 2. Benefits of Fibre Are Itself Uncertain,” *British Medical Journal* 300 (1990): 1337.

96 Alex Mold, *Making the Patient Consumer: Patient Organisations and Health Consumerism in Britain*, (Manchester: Manchester University Press, 2015).

97 E.H. Maclaren, “Authoritarianism in Diabetes,” *Diabetic Medicine* 6 (1989): 830.

98 R.B. Tattersall, “Authoritarianism in Diabetes,” *Diabetic Medicine* 6 (1989): 830-1. This was a quote from another clinician.

99 For instance: Tattersall and Jackson, “Social an Emotional Complications of Diabetes,” 271-85, 275-6; C.J.S. Probert, W. Maddison, J.M. Roland, “Diet, Diabetes and Male Chauvinism,” *British Medical Journal* 301 (1990): 1430-1.

100 R.D. Hill, *Diabetes Health Care: A Guide to the Provision of Health Care Services*, (London: Chapman and Hall, 1987).

101 Avner Offer, “Body Weight and Self-Control in the United States and Britain since the 1950s,” *Social History of Medicine* 14 (2001): 81.

102 Oral History Interview conducted with S. Sparrow by the author.

103 “Diets for Diabetics,” *British Medical Journal* 2 (1970): 66-7; D.K. McCulloch, R.D. Mitchell, J. Ambler and R.B. Tattersall, “Influence of Imaginative Teaching of Diet on Compliance and Metabolic Control in Insulin Dependent Diabetes,” *British Medical Journal* 287 (1983): 1858-61.

104 A.V. Thompson, H.A.W. Neil, M. Thorogood, G.H. Fowler, J.I. Mann, “Diabetes Mellitus: Attitudes, Knowledge and Glycaemic Control in a Cross-Sectional Population,” *Journal of the Royal College of General Practitioners* 38 (1988): 450-2; S.R. Heller, P. Clarke, H. Daly, I. Davis, D.K. McCulloch, S.P. Allison, R.B. Tattersall, “Group Education for Obese Patients with Type 2 Diabetes: Greater Success at Less Cost,” *Diabetic Medicine* 5 (1988): 552-6.

105 P. Ling, J.M. Lovesay, V.A. Mayon-White, A.H. Knight, “The Diabetic Clinic Dinosaur is Dying: Will Diabetic Day Units Evolve,” *Diabetic Medicine* 2 (1985): 163-5.

106 Valier and Bivins, “Organization, Ethnicity and the British National Health Service,” 37-64.

107 Peter Miller and Nikolas Rose, “Governing Economic Life,” *Economy and Society*19 (1990): 1-31.

108 Heller, et.al., “Group Education for Obese Patients with Type 2 Diabetes,” 552-6; Valier and Bivins, “Organization, Ethnicity and the British National Health Service,” 37-64.

109 Oral History Interview with Mo Linton.

110 Helen Zoe Veit, “‘So Few Fat Ones Grow Old’: Diet, Health and Virtue in the Golden Age of Rising Life Expectancy”, *Endeavour* 35 (2011): 91-8.

111 E.C. Spray, “Health and Medicine in the Enlightenment,” in Mark Jackson, ed., *The Oxford Handbook of the History of Medicine* (Oxford: Oxford University Press, 2011), 82-99.

